# Activation of Src, Fyn and Yes non-receptor tyrosine kinases in keratinocytes expressing human papillomavirus (HPV) type 16 E7 oncoprotein

**DOI:** 10.1186/1743-422X-10-79

**Published:** 2013-03-07

**Authors:** Anita Szalmás, Eszter Gyöngyösi, Annamária Ferenczi, Brigitta László, Tamás Karosi, Péter Csomor, Lajos Gergely, György Veress, József Kónya

**Affiliations:** 1 Department of Medical Microbiology, Medical and Health Science Center, University of Debrecen, Nagyerdei Krt. 98, Debrecen 4032, Hungary; 2 Department of Otolaryngology and Head and Neck Surgery, Medical and Health Science Center, University of Debrecen, Nagyerdei Krt. 98, Debrecen 4032, Hungary

**Keywords:** HPV 16, Oncoprotein, Non-receptor protein-tyrosine kinase, Src-family kinases

## Abstract

**Background:**

The Src family tyrosine kinases (SFK) are cellular regulatory proteins that influence cell adhesion, proliferation, invasion and survival during tumor development. Elevated activity of Src was associated with increased cell proliferation and invasivity in human papillomavirus (HPV)-associated malignancies; therefore, transduced human foreskin keratinocytes (HFK) were used to investigate whether SFK activation is a downstream effect of papillomaviral oncoproteins. Activation of ubiquitously expressed SFKs, namely Src, Yes and Fyn, was investigated in both proliferating and differentiating keratinocytes.

**Results:**

In proliferating keratinocytes, Src, Yes and Fyn mRNA levels were not affected by HPV 16 E6 or E7 oncoproteins, while at the protein level as detected by western blot, the presence of both E6 and E7 resulted in substantial increase in Src and Yes expression, but did not alter the high constitutive level of Fyn. Phospo-kinase array revealed that all ubiquitously expressed SFKs are activated by phosphorylation in the presence of HPV 16 E7 oncoprotein. Keratinocyte differentiation led to increased Yes mRNA and protein levels in all transduced cell lines, while it did not influence the Src transcription but resulted in elevated Src protein level in HPV16 E7 expressing lines.

**Conclusions:**

This study revealed that HPV 16 oncoproteins upregulate Src family kinases Src and Yes via posttranscriptional mechanisms. A further effect of HPV 16 E7 oncoprotein is to enhance the activating phosphorylation of SFKs expressed in keratinocytes.

## Background

The Src family cytoplasmic tyrosine kinases (SFK) are important signal transducers that are activated by many different classes of cell-surface receptors including receptor tyrosine kinases, cytokine receptors, and integrin extracellular matrix receptors [1-3]. This kinase family consists of 11 homologous non-receptor tyrosine kinases, namely Src, Fyn, Yes, Blk, Yrk, Frk, Fgr, Hck, Lck, Srm, and Lyn. Src, Fyn and Yes appear to be ubiquitously expressed in many different cell lineages, including keratinocytes of the squamous epithelium. SFKs are activated primarily via protein-protein interactions involving their SH2 domains, followed by a trans-autocatalytic phosphorylation of a conserved tyrosine residue in their catalytic domain [4,5]. SFKs interact with a network of intracellular pathways influencing cell adhesion, growth, movement and differentiation, therefore, SFKs may exhibit oncogenic activity when they become overactivated [6-8]. This overactivation is frequently detected in solid tumors where it has been associated with advanced disease stages and metastatic potential [9]. Therefore, small molecule inhibitors of SFKs were developed and tested successfully in some cancer types including ovarian, prostate, skin, hepatocellular, pancreatic, and non-small cell lung cancer for suppression of neoplastic cell proliferation and invasive capacity [10-15]. Recent studies have demonstrated elevated Src activity in cervical cancer tissues, and treatment with Src kinase inhibitors caused decreased cell motility and invasion abilities of cervical cancer cell lines [16-18]. Thus, Src inhibitors are considered as promising therapy molecules also for human cervical carcinomas [19]. However, the factors inducing SFK upregulation in cervical cancer, the second leading cause of death from cancer in women worldwide, are still to be clarified.

The major etiological factors of cervical cancer are human papillomaviruses (HPV), as nearly 100% of cervical cancers are positive for high-risk HPVs, HPV16 and HPV18 being the most prevalent genotypes detected in cervical carcinomas [20]. HPVs are considered as the most prevalent sexually transmitted infectious agents and are also frequently associated with other anogenital and oropharyngeal malignancies [21]. HPVs are small, non-enveloped DNA viruses that infect the cutaneous and mucosal epithelia with a life cycle tightly linked to the differentiation of the host epithelial cell [22]. Persistent high-risk HPV infection of epithelial cells results in permanent expression of the E6 and E7 viral oncoproteins, which play crucial roles in the induction and the maintenance of malignant transformation of the host cell [23,24]. The E6 and E7 oncoproteins of high-risk HPV viruses are able to reprogram their host cell regulatory pathways via associating with several signaling molecules involved in the regulation of cell-cycle progression and cellular differentiation, thereby promoting abnormal cell proliferation and neoplastic transformation [23,25]. This property of HPV oncoproteins raises the question whether the activation of SFKs in cervical cancer tissues and cell lines is a downstream effect of papillomaviral oncoproteins or it develops later during the oncogenic clonal selection.

In the present study, we investigated both the expression and the activation by phosphorylation of the ubiquitously expressed SFKs, namely Src, Yes, and Fyn in retrovirus transduced keratinocytes expressing HPV 16 E6, E7 or both oncoproteins under proliferating and differentiating culture conditions. Src and Yes expression was influenced differently by papillomaviral oncoproteins and differentiation, whereas the constitutively high Fyn expression was not affected by them. Nevertheless, the phosphorylation of the above SFKs was uniformly increased in the presence of E7 oncoprotein.

## Results

### Effect of HPV 16 E6 and E7 on the expression of Src family kinases in human keratinocytes

**Figure 1:**
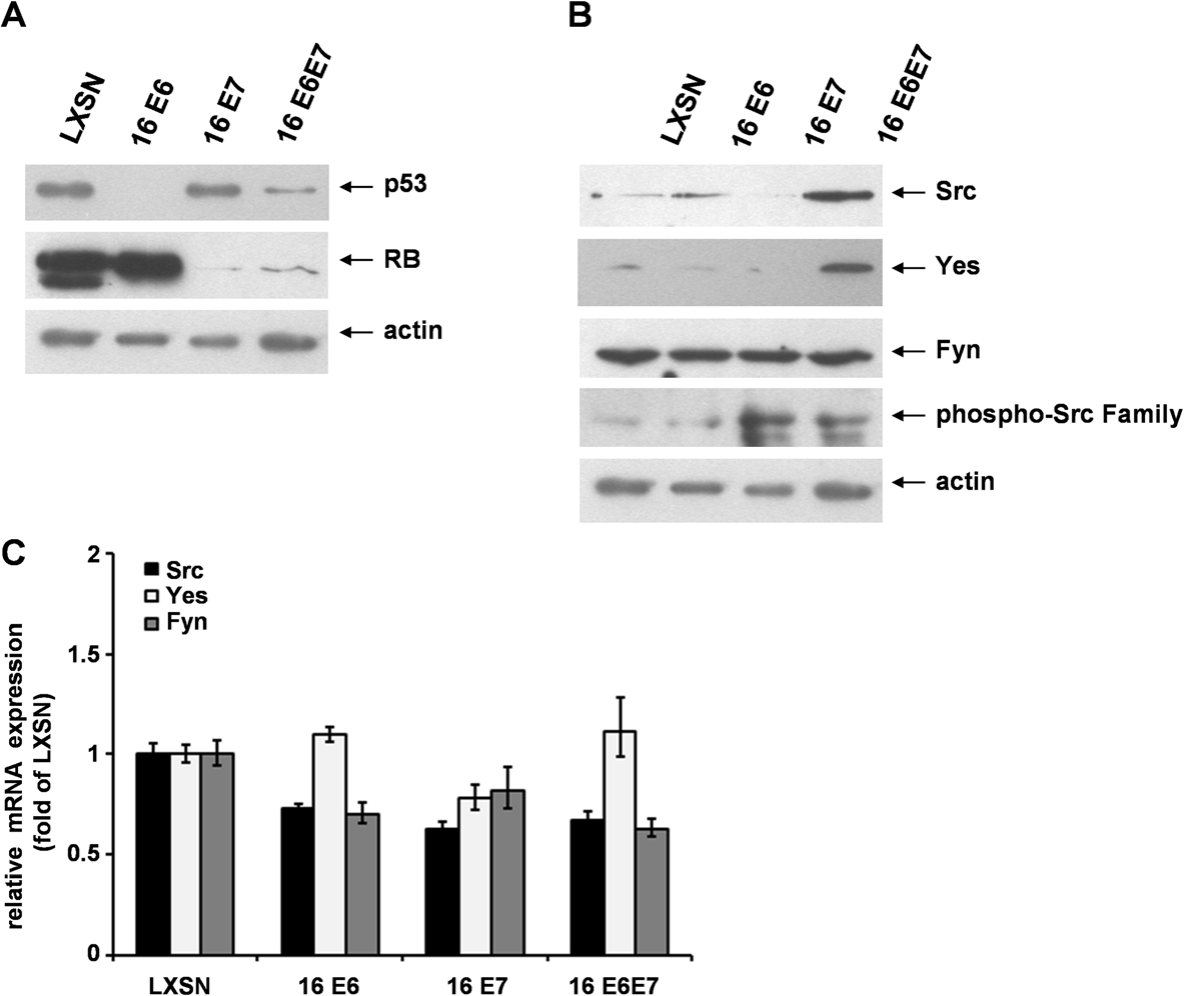
**Presence of HPV 16 E6 and E7 has different effect on SFK protein and mRNA expression in proliferating human keratinocytes.** (**A**) Western blot analysis of human foreskin keratinocytes (HFK) transduced with HPV 16 E6, E7 or both oncogenes. Levels of cellular p53 and Rb proteins are reduced in the presence of functionally active HPV 16 E6 and E7 oncoproteins, respectively. (**B**) Analysis of Src, Yes, and Fyn protein expression by western blot using antibodies specific for the native form of the kinases and for a C-terminal tyrosine residue that is phosphorylated upon activation. Actin was used as loading control. The results shown are representative of three independent experiments. (**C**) The effect of HPV 16 oncogenes on the relative mRNA expression of ubiquitously expressed SFKs in HFK cells. Real-time PCR analysis of Src, Yes, and Fyn was performed using RNA purified from HPV 16 E6 and/or E7 transduced HFK cells. The values were normalized against GAPDH mRNA and presented as relative levels with respect to the value of cells transduced by LXSN. The bars represent the mean ± SEM of two independent experiments, each with triplicate measurements. Two-tailed Student t test revealed no significant, HPV related alteration in any of the tested SFK mRNA levels.

Human foreskin keratinocytes (HFK) expressing HPV 16 oncoproteins were tested for the expression and the activity of SFKs. HFK cells were transduced by recombinant retroviruses carrying either the empty control vector (LXSN) or vectors encoding HPV 16 E6, E7 or both oncogenes as described previously [26]. The cell lines were cultured in serum-free medium to maintain proliferation. The presence of functionally active E6 and E7 oncoproteins in the studied cells was confirmed by Western blot analysis revealing altered expression of their main targets, the cellular p53 and Rb tumor suppressor proteins, respectively (Figure 1A). Substantial reduction of p53 protein level indicated that E6 transduced cell lines indeed expressed the functional oncoprotein. Analogously, the presence of functional E7 was indicated by decreased level of Rb protein.

In light of recent publications indicating that certain SFKs might be overactivated during the process of HPV-associated cervical carcinogenesis [16-18], we examined both the individual and the combined effects of HPV 16 E6 and E7 oncoproteins on the expression and activity of ubiquitously expressed SFKs, namely Src, Yes, and Fyn. Western blot analysis revealed that there was a significant increase in Src and Yes protein levels in the presence of both oncogenes, compared to the cells containing the empty vector or only one of the oncogenes (Figure 1B). A constitutively high level of Fyn protein was observed in all the studied cell lines suggesting that E6 and E7 oncoproteins have little, if any further effect on Fyn expression in human keratinocytes. Western blot analysis using phospho-Src antibody revealed that SFK activation by phosphorylation required E7 but was independent form E6 function. The applied antibody can detect both Src phosphorylated at Y416 and other SFKs phosphorylated at homologous tyrosine residues. Since both the steady state level and the activation state of two SFKs, Src and Fyn were affected by HPV 16 oncoproteins, we also quantitated the RNA transcripts of the studied SFKs. Nevertheless, the quantitative RT-PCR analysis could not reveal transcriptional mechanisms behind the different protein levels, the HPV 16 oncoproteins did not alter significantly the transcription of either SFK (Src, Yes) with inducible protein level, and the HPV related transcriptional pattern of Src was very similar to that of Fyn with constitutively high protein level (Figure 1C).

### Effect of HPV 16 E6 and E7 on the phosphorylation of Src family kinases in human keratinocytes

**Figure 2:**
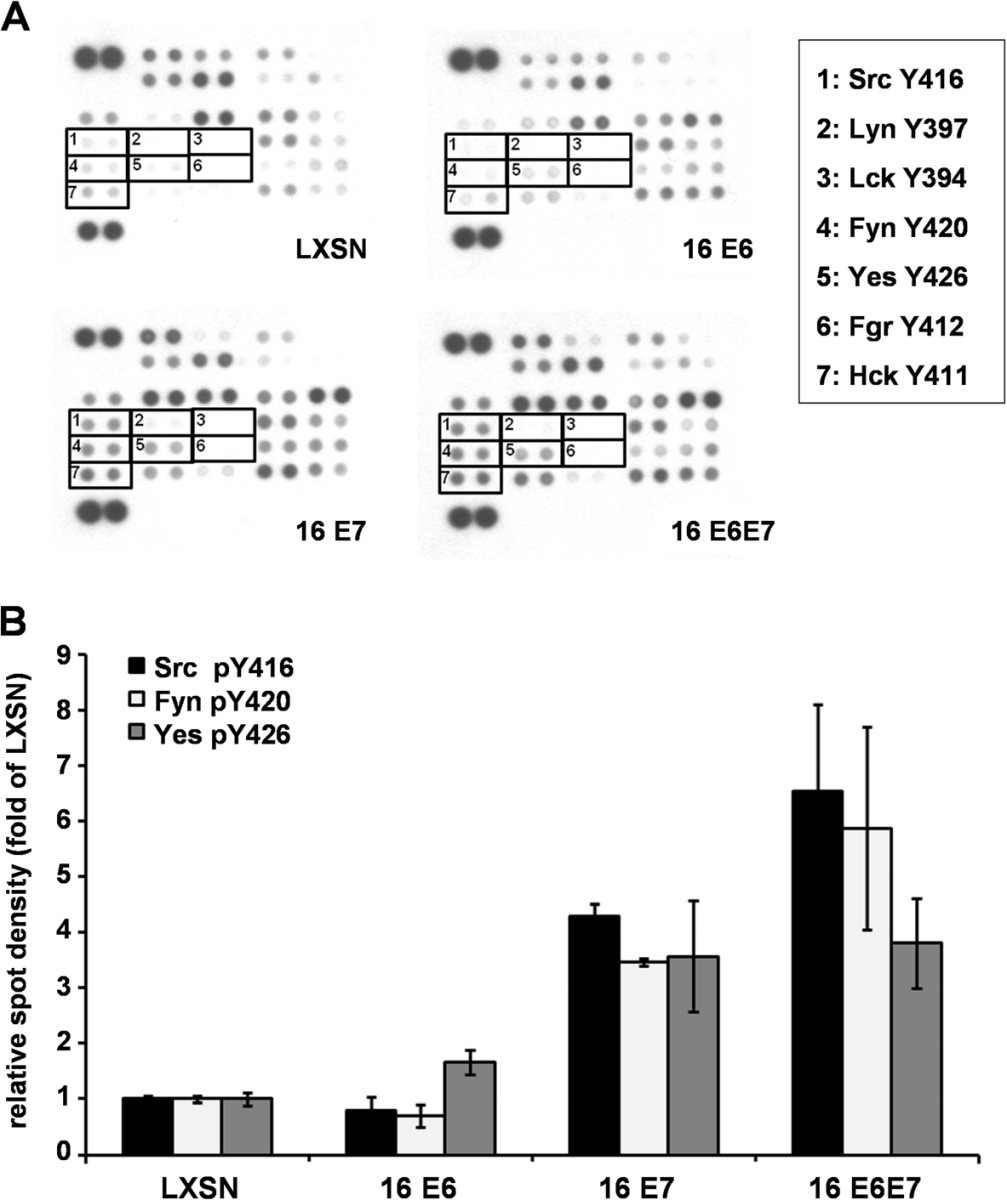
**Human phospho- kinase array to study the effect of HPV16 E6 and E7 on the phosphorilation state of SFKs.** (**A**) Whole cell lysates were analyzed with a human phospho-kinase array to detect phosporylation state of SFKs in HFK cells transduced with HPV 16 E6 or E7 or both oncogenes. Signals of tyrosine phosphorilated SFKs are indicated in the membranes, and intensity of corresponding spots are presented as bar graphs. (**B**) Spot-densities of phosphoproteins were quantified by using ImageJ software and compared to those of LXSN control spots. Data are presented as mean ± SEM of two independent experiments with duplicates.

Since western blot analysis revealed that the presence of HPV 16 E7 is associated with elevated SFK phosphorylation in HFK cells, we wished to evaluate the effect of E6 and E7 oncoproteins on the phosphorylation state of the individual members of the Src family on those tyrosine residues that are phosphorylated upon activation and therefore indicating increased kinase activity. To this end, human phospho-kinase array was performed from whole cell lysates. The results showed that in the presence of E7 there was a significant increase in the phosphorylation levels of all three ubiquitously expressed SFKs, while E6 had no effect on their activating phosphorylation when compared to control cells (Figure 2A-B).

**Figure 3:**
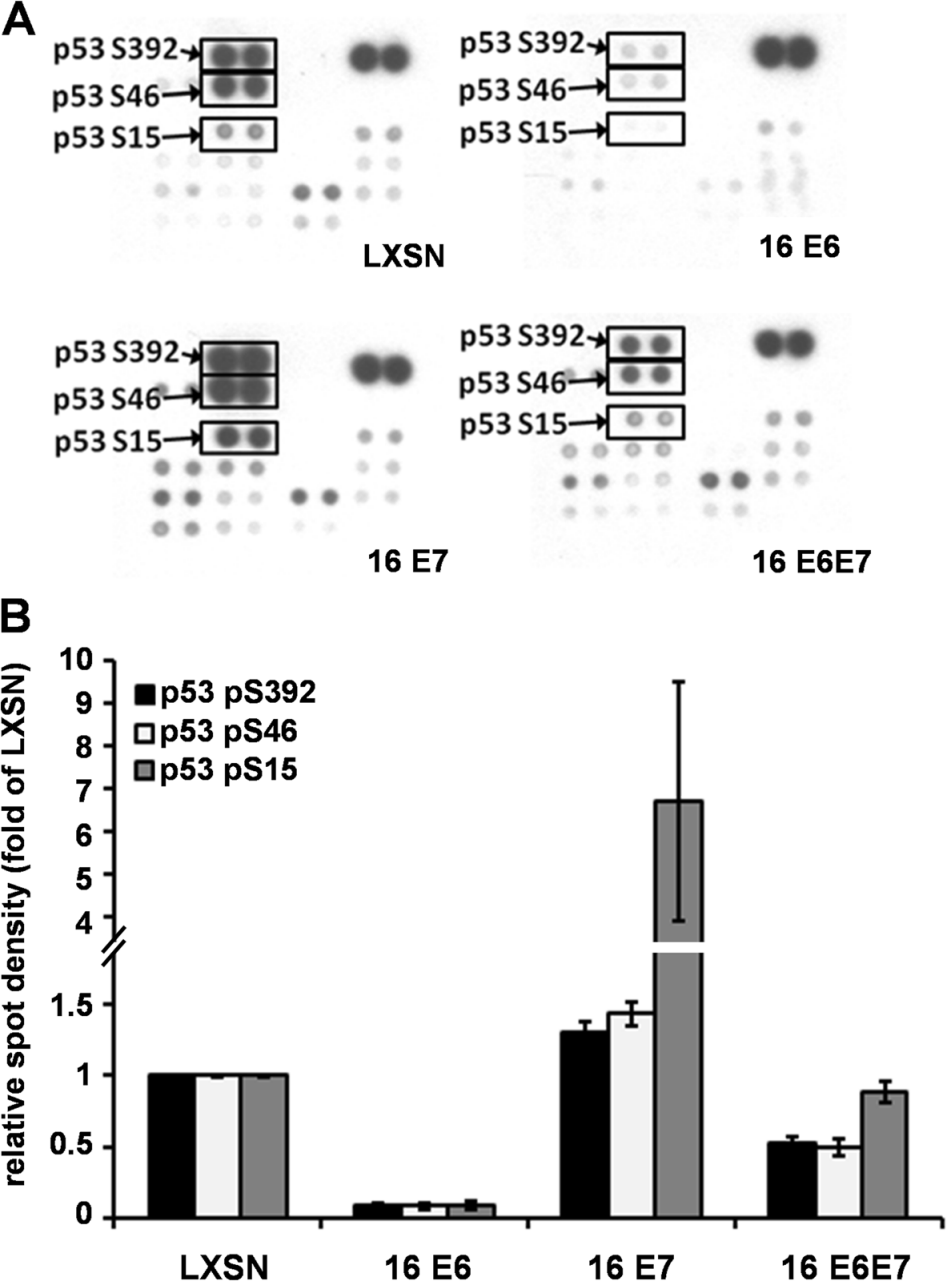
**Effect of HPV 16 E6 and E7 on the phosphorilation state of p53.** (**A**) p53 phosphorilation at serine residues S392, S46, and S15 detected by phosho-kinase array. Different phosphorilation variants of p53 are indicated by arrows in the membranes, and intensity of corresponding spots are presented as graphics (**B**). Spot-densities of phosphoproteins were quantified by using ImageJ software and compared to those of LXSN control spots. Data are presented as mean ± SEM of two independent experiments with duplicates.

The phospho-kinase array also provided further information on activating phosphorylation of relevant proteins such as p53 and four other SFKs. As expected, the level of p53 protein phosphorylated at serine residues S15, S46 or S392 increased in the presence of E7 oncoprotein and reflected well the p53 degradation in the presence of E6 (Figure 3A-B). The p53 phosphorylation pattern assisted the study in two ways: first, it further confirmed the eligibility of the transduced cell lines for papillomaviral functional analysis; second, it revealed that the capture based array could measure even the phosphorylated fraction of protein levels hardly or not detected by western blot.

The other extra information obtained from the phospho-kinase array was the detection of four other SFKs, namely Lyn, Lck, Fgr, and Hck, which tend to be expressed in cells of lymphoid origin. All transduced keratinocyte lines lacked activated Lyn, Lck and Fgr non-receptor tyrosine kinases, while some spot densities could be detected for activated Hck in the presence of E7 (Figure 2A). Hck mRNA could also be detected independently from papillomaviral oncoproteins (data not shown); however, the Hck protein level was below the detection limit of western blot suggesting a minor role for this member of the family in keratinocytes.

### Cellular differentiation alters expression of SFKs in keratinocytes expressing HPV16 E6, E7 or both oncoproteins

**Figure 4:**
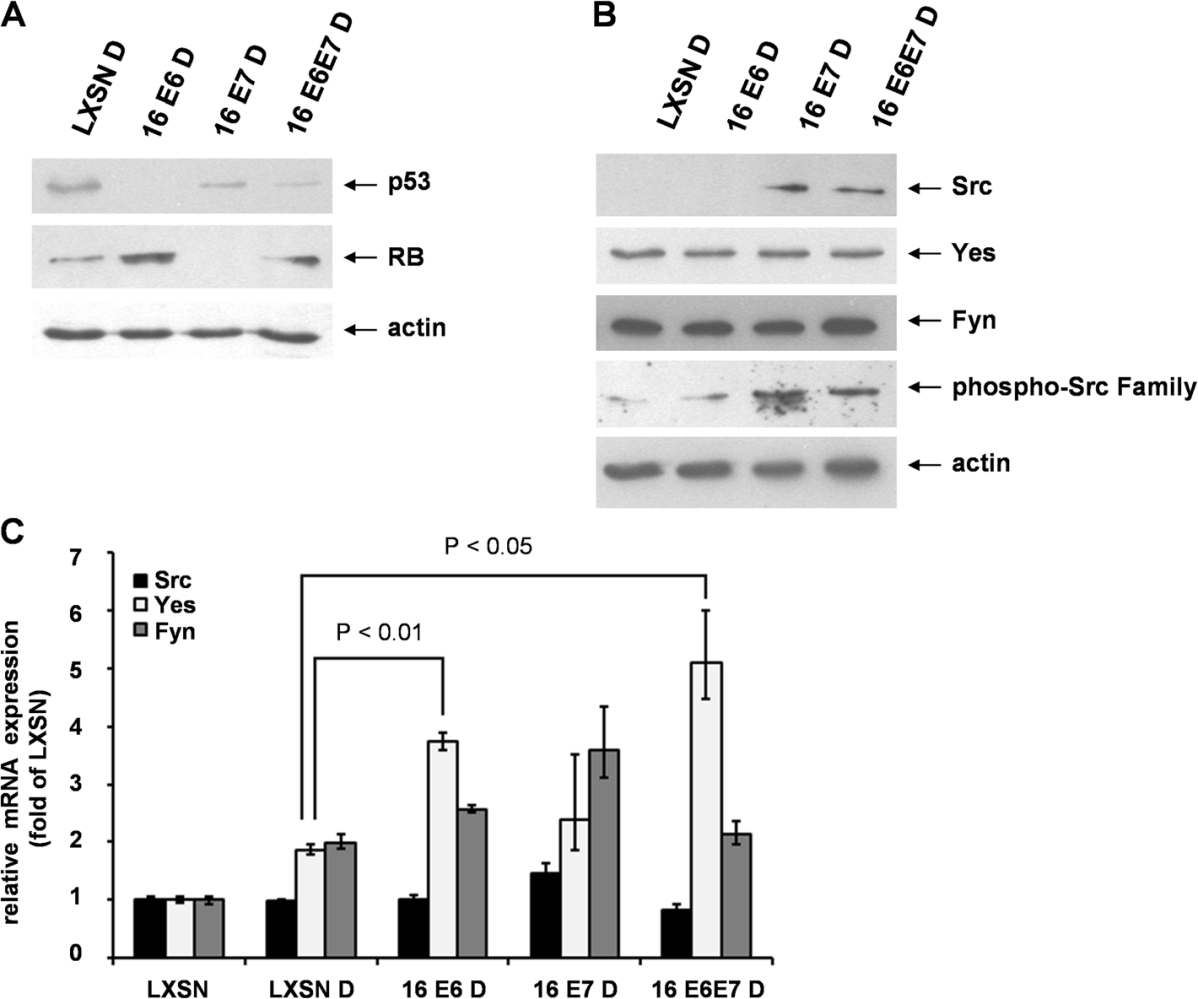
**Differentiation of human keratinocytes influences SFK protein and mRNA expression in transduced HFK cells.** (**A**) Western blot analysis of differentiating human foreskin keratinocytes (HFK) transduced with HPV 16 E6, E7 or both oncogenes. Cellular differentiation was induced by culturing cells in the presence of serum and high calcium. Levels of cellular p53 and Rb proteins are reduced in the presence of functionally active HPV 16 E6 and E7 oncoproteins, respectively. (**B**) Analysis of Src, Yes, and Fyn protein expression by western blot using antibodies specific for the native form of the kinases and for a C-terminal tyrosine residue that is phosphorylated upon activation. Actin was used as loading control. The results shown are representative of three independent experiments. (**C**) The effect of HPV 16 oncogenes on the relative mRNA expression of Src, Yes, and Fyn in differentiating HFK cells. Real-time PCR analysis was performed using RNA purified from the transduced HFK cells. The values were normalized against GAPDH mRNA and presented as relative levels with respect to the value of cells transduced by LXSN. The bars represent the mean ± SEM of two independent experiments, each with triplicate measurements. Two-tailed Student t-test was carried out for the analysis of mRNA expression levels to determine statistical significance (P < 0.05).

Next, we studied whether the effect of HPV 16 E6 and E7 oncoproteins on the expression and activity of Src, Yes and Fyn could be affected by cellular differentiation. Human keratinocytes transduced by HPV 16 E6, E7 or both oncogenes were cultured in the presence of serum and high calcium to induce cellular differentiation. In the tested cell cultures, successful initiation of cellular differentiation was confirmed as described previously [26]. First we confirmed that the activities of E6 and E7 oncoproteins were maintained in differentiating cells as well. Concomitant with the presence of E6 or E7, decreased levels of p53 or pRb proteins could be observed, respectively (Figure 4A).

In differentiating keratinocytes, Src protein expression was upregulated in a similar manner to that in proliferating keratinocytes except the presence of E7 alone was sufficient to induce upregulation (Figure 4B). Neither differentiation, nor the presence of papillomaviral oncoproteins resulted in significantly altered Src mRNA levels (Figure 4C). On the other hand, differentiation upregulated Yes protein expression to a level not influenced by papillomaviral oncoproteins (Figure 4B). Yes upregulation involved also transcriptional mechanisms, differentiation itself resulted in significant increase (P < 0.05) in Yes mRNA levels. Unlike in proliferating HFK lines, presence of E6 in the differentiating lines was significantly (P < 0.05) associated with increased Yes mRNA expression (Figure 4C). However, this transcriptional effect of E6 did not result in detectable alteration of Yes protein level (Figure 4B). Fyn protein level, likewise in proliferating HFK cells, was constitutively high and independent from papillomaviral oncoproteins (Figure 4B). Fyn mRNA level was also significantly increased (P < 0.05) in differentiating keratinocytes but it was not significantly altered in the presence papillomaviral oncoproteins (Figure 4C). Although differentiation of keratinocytes proved to act as a further regulatory mechanism for the expression of certain SFKs, it left SFK phosphorylation unaffected (Figure 4B).

## Discussion

This study identified Src family non-receptor tyrosine kinases (SFKs) that are activated in the presence of HPV 16 oncoproteins. Enhanced activity of SFKs has been detected in a wide range of malignancies including cervical cancer, where the most studied member of the family, Src was shown to be overactive based on the presence of activating phosphorylation at Y416 [5,7,8]. Besides Src, this kinase family involves two other ubiquitously expressed non-receptor tyrosine kinases, namely Yes and Fyn, which are expressed in various cell types including keratinocytes. Although Yes and Fyn have not yet been studied in cervical cancer, they are overactivated in several malignancies. For instance, Yes kinase has been associated with increased cell proliferation and invasion of human melanoma and colon carcinoma cells [9,27] and Fyn kinase has been implicated in the progression and metastasis of prostate cancer [14]. Since activation of SFKs occurs by phosphorylation at a C-terminal Y416 residue of Src and at homologous tyrosine residues of the other members of the family, therefore, in our experiments we also used a phospho-SFK specific antibody to assess the changes in the overall activity of the SFKs, then we refined the individual activation state of these kinases.

In this study, transduction of primary human keratinocytes ensured the detection of early downstream effects of the papillomaviral oncogenes in the natural host cells. The experiments were performed from 5 to 8 passages after transduction, therefore, our observations were probably unaffected by clonal expansion of any random genomic alteration. Primary human keratinocytes can also be driven towards differentiation, which leads to changes similar to those in the upper layers of the stratified squamous epithelium. Differentiating squamous cells lose their ability to proliferate and to replicate DNA which results in an intracellular condition targeted and reverted by papillomaviral oncoproteins. This mechanism ensures the effective viral replication in natural HPV infection [28,29]. As previously shown, the applied method was suitable to induce keratinocyte differentiation, thus, the differentiating keratinocyte cultures were considered to represent the upper layers, while the proliferating cultures to represent the basal layer of the squamous epithelium [26].

Cervical carcinogenesis has the unique feature of requiring papillomaviral i.e. exogenous oncoproteins from initiation to final stage. The E6 and E7 oncoproteins of the high-risk HPV types promote viral replication by interacting with cellular regulatory proteins [25]. More specifically, association of high-risk E6 with tumor suppressor protein p53 leads to the proteasomal degradation of p53 via recruitment of an ubiquitin ligase, E6-AP. In addition, p53-independent activities of E6 such as telomerase activation, association with PDZ proteins and other cellular target proteins may also contribute to the oncogenic activities of high-risk E6 proteins [30,31]. High-risk E7 oncoprotein is able to promote neoplastic transformation mainly by binding and subsequently promoting the proteasome-mediated degradation of the hypophosphorylated, growth suppressive form of the retinoblastoma tumor suppressor protein (pRb, p105) and the related pocket proteins p107 and pRb2/p130, thus, causing aberrant S-phase entry in cells that would have normally withdrawn from the cell division cycle. Besides pRb, E7 can also associate and interfere with the activities of multiple cellular factors, such as AP1, HDAC-1, PCAF, BRCA1, CDK, and ATM that contribute to the dysregulation of the cell cycle and apoptosis, and cause genomic instability [25,32]. In this study, the levels of p53 and pRb tumor suppressor proteins in the transduced cells were used to demonstrate the presence of functional E6 and E7, respectively.

The key role of papillomaviral oncoproteins in cervical carcinogenesis, the ubiquitous availability of Src, Fyn and Yes non-tyrosine receptor kinases and the observed Src activation in cervical cancer suggest a link between these important oncogenic factors and the papillomaviral oncoproteins. Indeed, increased Src Y416 phosphorylation has been observed in both established cell lines (SiHa, HeLa) and biopsies of cervical carcinoma origin [16]. The importance of SFK activation during cervical carcinogenesis is further supported by the notion that treatment with a small molecule Src kinase inhibitor or suppression of Src mRNA by siRNA treatment inhibited the proliferation of cervical cancer cell lines HeLa and SiHa [16]. Yasmeen and colleagues demonstrated that treatment of the above mentioned cervical carcinoma cell lines with Src kinase inhibitors significantly decreases cell motility and invasion abilities, as well [18]. Furthermore, nude mice xenograft experiments using HeLa cells showed that Src kinase inhibitors can also inhibit subcutaneous tumor growth significantly [16,17]. Despite the accumulating evidence regarding the importance of Src activation during cervical carcinogenesis, it is still not known whether the activation of Src and other SFKs in cervical cancer tissues and established cell lines is linked to papillomaviral oncoproteins or it develops later during the oncogenic clonal selection.

To investigate the involvement of high-risk HPV oncoproteins in SFK activation, first the effect of HPV 16 E6 and E7 oncoproteins was analyzed on SFK protein expression in transduced human primary keratinocytes, which revealed a heterogeneous response by Src, Yes and Fyn. In details, Src and Yes had similar requirement for both E6 and E7 to be upregulated in proliferating but not in differentiating keratinocytes. The latter condition itself made all transduced cell lines upregulate Yes protein expression with no further effect by papillomaviral oncoproteins. For Src upregulation, differentiation maintained the necessity of E7 activity but abrogated that of E6. Fyn protein expression was not influenced by the studied papillomaviral oncoproteins at all. Noteworthy, mRNA alterations could not be identified in the background of HPV 16 mediated SFK upregulation suggesting the importance of posttranscriptional regulatory mechanisms.

Despite the heterogenous effects on the protein expression, activation of Src, Yes and Fyn by phosphorylation was uniformly dependent on the presence of HPV 16 E7. Thus, the HPV 16 E7 oncoprotein, assisted also by the E6, might have dual effect on SFK activity. It can activate constitutively available non receptor tyrosine kinase Fyn and it can also elevate the intracellular level of others such as Src and Yes. Being available, Src, Yes and Fyn are uniformly activated by phosphorylation on Y416 and homologous tyrosine residues, respectively, in the presence of HPV 16 E7.

In this study, we observed HPV related alterations in the protein expression and activation of SFKs as a phenomenon. Underlying mechanisms can be proposed based on other studies. Our results on HPV mediated SFK activation are in agreement with a recent study where a kinase screen performed in HPV 16 E7 expressing RKO colorectal carcinoma cell lines revealed, that HPV 16 E7 expression results in substantially altered kinase requirements for viability of RKO cells [33]. The presence of HPV 16 E7 was proposed to relieve the requirement of certain kinases for RKO viability either by targeting pRb and causing cell cycle progression or by targeting other signal transduction pathways. Another finding on simian virus 40 large T antigen revealed that pRb binding viral oncoproteins can activate Src by phosphorylation via relieving the pRb control [34].

In addition to the multiple intracellular pathways affected by papillomaviral oncoproteins, one has to consider their effect also on transmembrane tyrosine kinase receptors, since HPV has been shown to upregulate EGF-R and the associated focal adhesion kinase as well [35,36]. The increased activity of growth factor receptors and the associated signaling molecules can also lead to SFK overactivation [37-40]. Nevertheless, a model system of culturing homogeneous cell population will more likely detect mechanisms confined to the intracellular compartments then exogenous receptor mediated effects requiring cognate ligand binding.

Recent studies focusing on the downstream effects of Src activation in cervical cancer suggest that our results describing the phenomenon can be verified by functional assays to assess the effect of HPV oncoproteins on migrating or invading properties of the host cell [16-18]. In this approach, either mutated HPV oncogenes coding oncoproteins with well characterized deficiencies or suppression of HPV oncogene expression by RNA interference might be used. Correlating the HPV related SFK alterations to epithelial structure and keratinocyte interactions in either organotypic raft cultures or xenograft studies can also support our findings in keratinocytes with calcium induced differentiation.

We believe the present study enlightened an important mechanism of papillomaviral oncoproteins which contributes to the development of malignant phenotype of the host epithelial cell. Since HPV oncogene expression is necessary to maintain the malignant phenotype of cervical cancer cells, the HPV related alterations such as the altered SFK expression or activity can be regarded as both initiating and maintaining oncogenic mechanisms.

## Conclusion

In addition to the high constitutive level of Fyn in keratinocytes, the E6 and E7 oncoproteins of HPV 16 increase the level of other Src family kinases such as Src and Yes via posttranscriptional mechanism. A further effect of HPV 16 E7 oncoprotein is to enhance the activating phosphorylation of all available SFKs. Taken together, our findings imply that high-risk HPV oncoproteins might influence the activation of SFKs during the development of HPV-associated malignancies, which should be taken into consideration during the development of treatment strategies with SFK inhibitors for these cancers.

## Materials and methods

### Cell cultures

Primary neonatal human foreskin keratinocytes (HFK) purchased from Invitrogen were transduced with the control LXSN retroviral vector or LXSN-based retroviral vectors expressing HPV16 E6, HPV16 E7 or both HPV16 E6 and E7 genes as described previously [26]. Infected HFK cells were either cultured in Defined Keratinocyte-Serum Free Medium containing < 0.1 mM calcium (DK-SFM, Invitrogen) to promote proliferation or induced to differentiate by culturing for 24 h in DMEM (Sigma) containing 1.8 mM calcium and 10% fetal calf serum (Gibco).

### Western blot

Proliferating and differentiating HFK cells were collected by trypsin treatment and whole cellular protein extracts were obtained by using RIPA lysis buffer (150 mM NaCl, 1% NP-40, 50 mM Tris–HCl pH 8.0, 0.5% Na-dezoxycholate, 0.1% SDS, 0.01% Na-azide, 1 mM EDTA, pH 7.4) supplemented with Complete EDTA-free Protease Inhibitor Cocktail (Roche), 1 mM NaF, and 1 mM Na_3_VO_4_. The total protein concentration of the lysates was estimated using Bradford protein assay. The extracts were mixed with Laemmli buffer, incubated at 95°C for 5 minutes, and electrophoresed on 10% SDS-polyacrilamide gel. 25 μg of each sample was loaded per lane. The separated proteins were electrotransferred onto nitrocellulose membrane (GE Healthcare) and after transfer, the membrane was blocked using 3% BSA in phosphate-buffered saline (pH 7.2) containing 0.05% Tween20 (PBST). Membrane was probed with mouse monoclonal anti-p53 (sc-126, Santa Cruz), mouse monoclonal anti-Rb (sc-102, Santa Cruz), mouse monoclonal anti-Src (sc-5266, Santa Cruz), mouse monoclonal anti-Yes (sc-48396, Santa Cruz), mouse monoclonal anti-Fyn (sc-271293, Santa Cruz), rabbit polyclonal anti-pY416 Src (2101, Cell Signaling), or rabbit polyclonal anti-actin (A2066, Sigma) primary antibodies diluted in 3% BSA in PBST. The blot was then incubated with a HRP-conjugated goat anti-mouse (sc-2005, Santa Cruz), or HRP-conjugated goat anti-rabbit (sc-2004, Santa Cruz) at a dilution of 1:10000 in 3% BSA in PBST for 1 h at room temperature. The signals were detected by using SuperSignal West Pico Chemiluminescent Substrate (Pierce) followed by exposure to X-ray film (Thermo Scientific).

### Phospho-kinase array

Protein phosphorylation was detected using the Human Phospho-Kinase Array Kit (Proteome Prolifer Array, R&D Systems). Total protein extracts were prepared from HFK cells transduced by recombinant retroviruses carrying either the control vector (LXSN) or vectors encoding HPV 16 E6, E7, or E6/E7. The signals were detected by using SuperSignal West Pico Chemiluminescent Substrate (Pierce) followed by exposure to X-ray film (Thermo Scientific). The obtained signals were quantified with ImageJ software.

### Quantitative real-time RT-PCR

Total RNA was isolated from cells using TRI reagent (Sigma) according to the manufacturer’s instructions and revers transcribed using the High Capacity cDNA Reverse Transcription Kit (Life Technologies). Quantitative real-time PCR was performed on 7500 Real Time PCR System (Life Technologies) using TaqMan Gene Expression Master Mix and Assays according to the manufacturer’s recommendations (all from Life Technologies) at a total volume of 20 μl. The applied TaqMan Gene Expression Assays were for Src (SRC; Hs01082246_m1), Yes (YES; Hs01080041_g1), Fyn (FYN; Hs00941600_m1), Hck (HCK; Hs00176654_m1), and glyceraldehyde 3-phosphate dehydrogenase (GAPDH; 0711024) as endogenous control. Each PCR reaction was performed in triplicate at least three times.

### Statistical analysis

For the analysis of real-time RT-PCR results, the comparative Ct method was used to obtain the Relative Quantification (RQ) values (7500 System SDS Software, version 1.4). Statistical anlysis of gene expression data was performed with two-tailed Student t test with P < 0.05 considered as statistically significant.

To analyse the results of the phospho-kinase arrays, mean and SEM of standardized spot densities were calculated (from two independent experiments with duplicates).

## Abbreviations

AP1: Activator protein 1; ATM: Ataxia telangiectasia mutated; BRCA1: Breast cancer type 1; CDK: Cyclin-dependent kinase; DK-SFM: Defined Keratinocyte-Serum Free Medium; DMEM: Dulbecco’s modified Eagle’s medium; EDTA: Ethylenediaminetetraacetic acid; HDAC: Histone deacetylase; HFK: Human foreskin keratinocyte; HPV: Human papillomavirus; HRP: Horseradish peroxidase; NaF: Sodium fluoride; Na3VO4: Sodium orthovanadate; PBS: Phosphate buffered saline; PBST: Phosphate-buffered saline–Tween; PCAF: P300/CBP-associated factor; RB: Retinoblastoma protein; SDS: Sodium dodecyl sulfate; SEM: Standard error of mean; SFK: Src family kinase.

## Competing interests

The authors declare that they have no competing interests.

## Authors’ contributions

AS designed and performed the experiments and drafted the manuscript. EG and GV provided and characterized the human cells used in the experiments. AF, BL, and PC helped in experiments. LG and TK contributed to writing the manuscript. JK participated in the financial support and contributed to writing the manuscript. All authors have read and approved the final manuscript.

## Authors’ information

Department of Medical Microbiology, Medical and Health Science Center, University of Debrecen, H4032 Debrecen, Nagyerdei krt. 98., Hungary, Tel: +36-52-255425, Fax: +36-52-255424.
